# The impact of septicemia occurring during hospitalization for renal transplantation procedures on outcomes in adults in United States

**DOI:** 10.1371/journal.pone.0179466

**Published:** 2017-06-13

**Authors:** Veerajalandhar Allareddy, Sankeerth Rampa, Alexandre T. Rotta, Veerasathpurush Allareddy

**Affiliations:** 1 Division of Pediatric Critical Care, Stead Family Children’s Hospital, University of Iowa, Iowa City, Iowa, United States of America; 2 College of Public Health, Department of Health Services and Research, University of Nebraska Medical Center, Omaha, Nebraska, United States of America; 3 Division of Pediatric Critical Care, Rainbow Babies & Children’s Hospital, Case Western Reserve University, Cleveland, Ohio, United States of America; 4 College of Dentistry, University of Iowa, Iowa City, Iowa, United States of America; National Yang-Ming University, TAIWAN

## Abstract

**Introduction:**

Immediate success rates of renal transplantation (RT) procedures are generally very high. National estimates of the impact of post-operative complications, specifically, septicemia occurring during hospitalization for RT’s on outcomes is unclear. We sought, to examine the prevalence of septicemia in patients having renal transplantation procedures and to quantify the impact of septicemia on in-hospital mortality (IHM), length of stay (LOS), and hospital charges (HC).

**Materials and methods:**

We performed a retrospective analysis of the Nationwide Inpatient Sample (NIS) for the years 2004 to 2010. All patients aged ≥18 years who underwent RT were selected. Association between occurrence of septicemia and outcomes (IHM, HC and LOS) was examined by multivariable linear and logistic regression models with adjustments for patient and hospital level confounders.

**Results:**

During the study period, 113,058 patients underwent RT, and, of these, 2459 (2.2%) developed septicemia. Characteristics included mean age (50 years), gender (males, 60%) and race (whites, 54%). Majority of RT’s were performed in teaching (96%) and large institutes (85.5%). Outcomes for patients with septicemia and without septicemia, included: IHM (12.9% vs. 0.4%), discharge routinely (42.4% vs. 82.6%), mean HC ($528,980 vs. $182,165), mean LOS in days (35.2 vs 7.3), respectively, Those who developed septicemia were associated with significantly higher HC (estimate: 0.8357, 95% CI: {0.7636–0.9077}, increase of $ 247,081 from mean, p<0.0001), longer LOS (1.2116{1.1015–1.3216}, increase of 18.7 days form mean, p<0.0001) and higher IHM (Odds ratio = 31.33; {20.25–48.48}, p < 0.0001) compared to their counterparts. Increasing age (OR = 1.02 {1.01–1.02}, p<0.0001) and increase in co-morbid burden (OR = 1.57 {1.42–1.74}, p<0.0001) were associated with higher risk for developing septicemia.

**Conclusions:**

Despite advances in medical/surgical care, septicemia is not an uncommon complication in patients having renal transplantation procedures and is associated with poor outcomes. Increasing age and co-morbid burden are independent predictors of occurrence of septicemia.

## Introduction

Renal transplantation, either from cadaveric or living donor, is considered the treatment of choice for most adult patients with end-stage renal disease. Successful renal transplantation provides substantially longer survival, improved quality of life and is more cost effective than maintenance dialysis [1–6]. Per the Organ Procurement and Transplantation Network of the U.S. Department of Health and Human Services, as of September 2014, there are more than 123,175 people waiting for lifesaving organ transplants, including **101,170** who await kidney transplants in the United States [7]. In the year 2013, **16,896** kidney transplants took place in the U.S [8]. Because of the relative scarcity of donated kidneys and the growing waitlist for transplantation, patient survival after renal transplantation is viewed as a quality measure of adequacy of candidacy selection and perioperative management. To optimize outcomes, the Organ Procurement and Transplantation Network proposed an allocation system for deceased donor kidneys using two metrics to allocation- namely, the kidney donor profile index (KDPI) [9] aimed at identifying the high quality kidneys, and the estimated post-transplant score (EPTS) based on the length of time on dialysis, any prior organ transplant, diabetic status and age. The KDPI and the EPTS are used to match the best 20% of donor kidneys to 20% of adult candidates with the longest EPTS [10].

Advances in medical and surgical care over the past six decades have led to a low complication rate following renal transplantation [11–12]. Immediate post-operative common complications in renal transplant recipients include vascular (thrombosis, stenosis), hemorrhagic, ureteral (leaks, stenosis) or infection [12]. Infection is a major cause of morbidity and mortality in any surgical cohort [13]. Healthcare-associated infection (HAI) in the renal transplant recipients could occur due to post-operative pneumonia, urinary tract infections, surgical site infections, catheter associated infections and/or other types of infections (such as C difficile). HAI of any cause is perceived as a major public health issue and is marker of quality of care delivered in United States [14, 15].

The impact of post-operative complications occurring during hospitalization for renal transplantation, especially infection, is unclear at a national level. The objectives of the present study are twofold. To estimate the prevalence of septicemia of ***any cause*** in adult patients who underwent renal transplantation procedures in United States. Further, we sought to quantify the impact of occurrence of septicemia on outcomes, specifically, in-hospital mortality, hospital charges and length of stay. We hypothesize that a mix of patient level factors predicts the occurrence of septicemia in this surgical cohort of patients.

## Materials and methods

### Design and description of Nationwide Inpatient Sample database

We performed a retrospective analysis of the Nationwide Inpatient Sample (NIS) database for the years 2004 to 2010. The NIS is the largest all-payer inpatient database in the United States. It is a part of the Healthcare Cost and Utilization Project (HCUP) sponsored by the Agency for Healthcare Research and Quality (AHRQ) [16]. The sampling frame of NIS is based on hospital location (rural or urban), hospital geographic region, bed-size of hospital, teaching status, and ownership/control. NIS provides discharge information on close to 40 million hospitalizations which approximates to 97% of all hospital discharges occurring in the United States annually. A multitude of data elements including primary and secondary diagnoses, co-morbidity measures, procedures performed during hospitalization, discharge status of patients, patient demographic information(including age, gender, race, and insurance status), type of admission(elective or emergency/urgent), hospitalization charges, and length of stay in hospital are available in the NIS.

### Institutional review board approval and data user agreement

The present study was exempt of institutional review board (IRB) approval by the University of Iowa IRB. The Federal Regulations 45 CFR 46.101 (b) states that *“research involving the collection or study of existing data*, *documents*, *records*, *pathological specimens*, *or diagnostic specimens*, *if these sources are* ***publicly available*** *or if the information is recorded by the investigator in such a manner that* ***subjects cannot be identified***, *directly or through identifiers linked to the subjects”*. Per this regulation such studies are permitted to be classified as research that is “exempt” from IRB full or expedited review. This present study was a retrospective analysis of hospital based discharge dataset that is **available** for purchase from AHRQ.

We completed a data user agreement with HCUP-AHRQ and obtained the NIS data sets. According to the data-user agreement, individual table cell counts of 10 or lower cannot be presented to preserve patient confidentiality. Consequently, these data were not reported in our study and are represented by the designation DS, for discharge information suppressed.

### Case selection

All adult patients (age ≥ 18 years) who had a renal transplantation procedure were selected for analysis. The ICD-9-CM procedural codes used to identify renal transplantation procedures included renal auto transplantation (ICD-9-CM procedure code of 55.61) and other kidney transplantation (55.69) [17]. In this surgical cohort those who developed septicemia during the **present hospitalization** were identified using ICD-9-CM codes. The ICD-9-CM diagnosis codes used to identify septicemia included Systemic inflammatory response syndrome, unspecified (995.90), Systemic inflammatory response syndrome due to infectious process without acute organ dysfunction (995.91), Systemic inflammatory response syndrome due to infectious process with acute organ dysfunction (995.92), Systemic inflammatory response syndrome due to non-infectious process without acute organ dysfunction (995.93), Systemic inflammatory response syndrome due to non-infectious process with acute organ dysfunction (995.94), Streptococcal septicemia (038.0), Staphylococcal septicemia, unspecified (038.10), Methicillin susceptible Staphylococcus aureus septicemia (038.11), Methicillin resistant Staphylococcus aureus septicemia (038.12), other staphylococcal septicemia (038.19), Pneumococcal septicemia [Streptococcus pneumoniae septicemia] (038.2), Septicemia due to anaerobes (038.3), Septicemia due to other gram-negative organisms—Gram-negative organism, unspecified (038.40), Septicemia due to other gram-negative organisms—Hemophilus influenzae (038.41), Septicemia due to other gram-negative organisms—Escherichia coli (038.42), Septicemia due to other gram-negative organisms—Pseudomonas (038.43), Septicemia due to other gram-negative organisms—Serratia (038.44), Septicemia due to other gram-negative organisms—Other (038.49), other specified septicemias (038.8), unspecified septicemia (038.9), herpetic septicemia (054.5), and bacteremia (790.7) [17].

### Outcome variables

The primary outcome variables of interest were in-hospital mortality, hospitalization charges, and hospital length of stay. All hospitalization charges were adjusted to year 2010 US dollar values using the hospital care inflation rates obtained from the Bureau of Labor Statistics [18].

### Independent variables

The main independent variables of interest were the occurrence of different types of septicemia. The demographic variables examined included sex, race, type of admission, insurance status, and co-morbid burden. The NIS co-morbid severity files were used to estimate the co-morbid burden. The NIS severity files examine 29 different co-morbid conditions including AIDS, alcohol abuse, deficiency anemias, rheumatoid arthritis/collagen vascular diseases, chronic blood loss anemia, congestive heart failure, chronic pulmonary disease, coagulopathy, depression, diabetes—uncomplicated, diabetes—with chronic complications, drug abuse, hypertension, liver disease, lymphoma, fluid and electrolyte disorders, metastatic cancer, neurological disorders, obesity, paralysis, peripheral vascular disorders, psychoses, pulmonary circulation disorders, renal failure, solid tumor without metastasis, peptic ulcer disease excluding bleeding, valvular disease, and weight loss. The occurrence of each of these conditions was summed to compute the co-morbid burden.

### Analytical approach

The baseline characteristics between those who developed septicemia and those who did not develop septicemia were examined by simple logistic (for categorical data) and simple linear (for continuous data) regression models. The association between the occurrence of septicemia and in-hospital mortality was examined by a multivariable logistic regression model. Odds ratios and 95% confidence intervals were computed in the logistic regression model. The multivariable logistic regression model fitness was assessed by the Hosmer and Lemeshow Goodness of Fit test [19]. The Chi-square value for this model was 7.11 and the p-value was 0.52. This indicates that the model fitness was good.

Multivariable linear regression models were used for hospital charges and length of stay in hospital. Since hospitalization charges and length of stay data were skewed, these were log transformed and used as dependent variables in the regression models. The distribution of the log transformed data was assessed by examining skewness, kurtosis and percentile distributions and then the multivariable linear regression models were fit on the log transformed data. The effects of age, sex, race, insurance status, co-morbid burden, teaching status of hospital, and hospital region were adjusted in the multivariable regression models. Taylor linearization method was used to fit the regression models [20]. Hospital stratum was used as the stratification variable. The unit of analysis was each individual hospitalization. The effects of clustering of outcomes were adjusted in the regression models. Further, a multivariable logistic regression model was used to examine the factors associated with the development of septicemia. This model also had a good fit (Hosmer and Lemeshow Goodness of Fit—chi-square value is 10.42 and p-value is 0.24). Since three outcomes (hospital charges, length of stay, and in-hospital mortality) were compared between those with and without septicemia, we conducted Bonferroni adjustments to account for potential Type 1 errors associated with multiple outcomes assessment. For the in-hospital mortality, hospital charges, and length of stay models, we deemed a p-value of <0.01 to be statistically significant (accounted for Bonferroni corrections). A p-value of <0.05 was deemed to be statistically significant for the multivariable logistic regression model predicting septicemia and for all simple logistic/linear regression models examining differences in distribution of baseline characteristics between those with and without septicemia. All statistical tests were two sided. Statistical analyses were conducted using SAS Version 9.3(SAS Institute, Cary, NC) and SUDAAN Version 10.0.1(Research Triangle Institute, NC) software.

## Results

During the study period, a total of 113,058 patients had a renal transplant procedure. Amongst this cohort, 2.2% developed septicemia (Table 1). The frequently occurring types of septicemia were: Systemic inflammatory response syndrome due to infectious process with acute organ dysfunction (0.7%), bacteremia (0.7%), unspecified septicemia (0.7%), and systemic inflammatory response syndrome due to infectious process without acute organ dysfunction (0.4%). Characteristics of patients who underwent the renal transplant procedures are summarized in Table 2. Overall, close to 60.8% of all patients were males. The mean age of those who did not develop septicemia was 50 years (compared to 52.9 years for those who developed septicemia). About 54% of all patients were white. Those who developed septicemia were more often hospitalized on an emergency/urgent basis (55.8% for those who developed septicemia versus 45.7% for those who did not develop septicemia). A vast majority of patients had some form of insurance. Those who developed septicemia had a higher co-morbid burden than those who did not develop septicemia (91.5% of those who developed septicemia had at least one chronic co-morbid condition while 83% of those that did not develop septicemia had at least one chronic co-morbid condition). The majority of procedures were performed in teaching hospitals and large hospitals.

**Table 1:** Types of septicemia in patients that had kidney transplantation procedures.

Type of Septicemia (ICD-9-CM Code)	% of All Transplant Procedures (N = 113,058)
Systemic inflammatory response syndrome, unspecified (995.90)	122 (0.1%)
Systemic inflammatory response syndrome due to infectious process without acute organ dysfunction (995.91)	487 (0.4%)
Systemic inflammatory response syndrome due to infectious process with acute organ dysfunction (995.92)	803 (0.7%)
Systemic inflammatory response syndrome due to non-infectious process without acute organ dysfunction (995.93)	59 (0.05%)
Systemic inflammatory response syndrome due to non-infectious process with acute organ dysfunction (995.94)	20 (0.01%)
Streptococcal septicemia (038.0)	138 (0.1%)
Staphylococcal septicemia, unspecified (038.10)	30 (0.03%)
Methicillin susceptible Staphylococcus aureus septicemia (038.11)	59 (0.05%)
Methicillin resistant Staphylococcus aureus septicemia (038.12)	DS
Other staphylococcal septicemia (038.19)	65 (0.06%)
Pneumococcal septicemia [Streptococcus pneumoniae septicemia] (038.2)	DS
Septicemia due to anaerobes (038.3)	20 (0.01%)
Septicemia due to other gram-negative organisms—Gram-negative organism, unspecified (038.40)	56 (0.05%)
Septicemia due to other gram-negative organisms—Hemophilus influenzae [H. influenzae] (038.41)	0
Septicemia due to other gram-negative organisms—Escherichia coli [E. coli] (038.42)	141 (0.1%)
Septicemia due to other gram-negative organisms—Pseudomonas (038.43)	75 (0.07%)
Septicemia due to other gram-negative organisms—Serratia (038.44)	39 (0.03%)
Septicemia due to other gram-negative organisms—Other (038.49)	78 (0.07%)
Other specified septicemias (038.8)	26 (0.02%)
Unspecified septicemia (038.9)	757 (0.7%)
Herpetic septicemia (054.5)	0
Bacteremia (790.7)	792 (0.7%)
Any of the above	2459 (2.2%)

**Table 2:** Characteristics of patients that had kidney transplantation procedures.

Characteristic		Did not Develop Septicemia (N = 110,599)	Developed Septicemia (N = 2,459)	p-value**
Sex	Male	60.8%	59.3%	0.55
	Female	39.2%	40.7%	
Race*	White	54.1%	54.1%	0.98
	Black	21.7%	23.1%	0.52
	Hispanic	15.2%	15%	0.81
	Asian/Pacific Islander	4.9%	4.7%	0.86
	Native American	1.2%	DS	0.09
	Other Races	2.8%	2.8%	0.92
Type of Admission	Emergency/Urgent	45.7%	55.8%	0.01
	Elective	54.3%	44.2%	
Insurance Status	Medicare	58.4%	59.7%	0.59
	Medicaid	3.8%	6.8%	<0.001
	Private Insurance	35%	28.8%	0.01
	Uninsured	0.6%	0.9%	0.46
	Other Insurances	2.1%	3.7%	0.02
Comorbid Burden	0	17%	8.5%	<0.0001
	1	29.4%	16.1%	
	2	26.1%	20.5%	
	3	15.8%	18.2%	
	4	7.2%	13.6%	
	5	2.8%	8.6%	
	6	1%	5.9%	
	≥ 7	0.5%	8.5%	
Hospital Bed Size	Small	1.4%	4.1%	0.05
	Medium	13.1%	12.5%	0.86
	Large	85.5%	83.4%	0.47
Hospital teaching status	Non-teaching	4%	3.4%	0.95
	Teaching	96%	96.6%	
Hospital Region	Northeast	16%	14%	0.49
	Midwest	22.8%	24.3%	0.62
	South	32.9%	34.6%	0.70
	West	28.2%	27.1%	0.72
Age	Mean in years	50 years	52.9 years	<0.0001
	Standard deviation	13 years	12.7 years	
	25^th^ Percentile	39.8 years	44 years	
	Median in years	50.5 years	53.6 years	
	75^th^ Percentile	59.5 years	62.3 years	
**Disposition Status**	Routine discharge	82.6%	42.4%	<0.0001
	Transferred to another short term acute care hospital	0.05%	0.8%	<0.0001
	Transferred to long term care facilities (e.g. skilled nursing facilities)	2%	23.2%	<0.0001
	Home health care	14.9%	20.8%	0.02
	Discharged against medical advice	0.04%	0	-
	Died in hospital	0.4%	12.9%	<0.0001
**Outcome**	**Measure**			
Hospital Charges	Mean	$182,165	$528,980	<0.0001
	Standard deviation	$113,181	$381,865	
	25^th^ Percentile	$119,072	$245,176	
	Median	$151,836	$398,857	
	75^th^ Percentile	$212,043	$683,434	
Length of Stay	Mean	7.3 days	35.2 days	<0.0001
	Standard deviation	6.7 days	36 days	
	25^th^ Percentile	4 days	12.7 days	
	Median	5.3 days	23.2 days	
	75^th^ Percentile	7.5 days	43.8 days	

*Information on race was available for 90,281 patients who did not develop septicemia and 2,013 patients who developed septicemia.

**Simple logistic regression models were used for categorical variables and simple linear regression models were used for continuous variables.

Disposition status of patients following the renal transplant procedure is summarized in Table 2. Most patients (82.6%) who did not develop septicemia were discharged routinely (compared to 42.4% of those who developed septicemia). In-hospital mortality rate was 12.9% among those who developed septicemia (compared to 0.4% in those who did not). Hospital charges and length of stay data are presented in Table 2. The mean charge for those who did not develop septicemia was $182,165 (median is $151,836) while the mean charge for those who developed septicemia was $528,980 (median is $398,857). The mean length of stay in hospital for those who did not develop septicemia was 7.3 days (median is 5.3 days) while the mean length of stay for those who developed septicemia was 35.2 days (median is 23.2 days).

Results of the multivariable regression models examining the occurrence of septicemia and hospital charges/length of stay/in-hospital mortality are summarized in Tables 3 to 5. Following adjustment for the effects of age, sex, race, insurance status, co-morbid burden, teaching status of hospital, and hospital region, those who developed septicemia were associated with significantly higher hospital charges ($247,081 more than mean, p<0.0001), longer length of stay in hospital (18.7 days more than mean, p<0.0001), and higher odds for in-hospital mortality (OR = 31.33, 95% CI = 20.25–48.48, p<0.0001).

**Table 3:** Summary of results from multivariable linear regression model examining the impact of septicemia on hospital charges.

Independent Variables		Parameter Estimate from Regression Model (95% CI)	Change from Mean	p-value
Septicemia	Yes	0.8357 (0.7636–0.9077)	$247,081	<0.0001
	No	Reference		
Age	Each 1 year increase	0.0005 (-0.0003–0.0012)	$95	0.22
Sex	Female	-0.0232 (-0.0372 –-0.0092)	-$4337	0.001
	Male	Reference		
Co-morbid burden	Each 1 unit increase	0.0945 (0.0797–0.1094)	$18,744	<0.0001
Race	Black	0.1093 (0.0560–0.1627)	$21,844	0.0001
	Hispanic	0.0698 (0.0106–0.1291)	$13,673	0.02
	Asian/Pacific Islander	0.0394 (-0.0216–0.1004)	$7,600	0.20
	Native American	-0.2305 (-0.3344 –-0.1267)	-$38,934	<0.0001
	Other Race	0.0194 (-0.0717–0.1106)	$3,705	0.67
	White	Reference		
Insurance status	Medicare	0.0322 (-0.1023–0.1667)	$6,189	0.64
	Medicaid	0.0961 (-0.0404–0.2327)	$19,077	0.17
	Private	-0.0587 (-0.1826–0.0652)	-$10782	0.35
	Other insurance	-0.2319 (-0.4014 –-0.0625)	-$39,144	0.008
	Uninsured	Reference		
Teaching status of hospital	Teaching	-0.2096 (-0.4117 –-0.0075)	-$35,762	0.04
	Non-teaching	Reference		
Geographic Region	Northeast	-0.1987 (-0.3873 –-0.0100)	-$34,081	0.04
	Midwest	-0.0091 (-0.1715–0.1532)	-$1,713	0.91
	South	-0.2336 (-0.3916 –-0.0756)	-$39,399	0.004
	West	Reference		

**Table 4:** Summary of results from multivariable linear regression model examining the impact of septicemia on length of stay.

Independent Variables		Parameter Estimate from Regression Model (95% CI)	Change from Mean	p-value
Septicemia	Yes	1.2116 (1.1015–1.3216)	18.7 days	<0.0001
	No	Reference		
Age	Each 1 year increase	0.0016 (0.0009–0.0024)	0.01 days	<0.0001
Sex	Female	-0.0152 (-0.0328–0.0024)	-0.1 days	0.09
	Male	Reference		
Co-morbid burden	Each 1 unit increase	0.1176 (0.0994–0.1359)	1 day	<0.0001
Race	Black	0.0566 (0.0199–0.0933)	0.5 days	0.003
	Hispanic	0.0034 (-0.0453–0.0521)	0.03 days	0.90
	Asian/Pacific Islander	-0.0105 (-0.0660–0.0450)	-0.08 days	0.71
	Native American	-0.0902 (-0.2597–0.0794)	-0.7 days	0.29
	Other Race	-0.0042 (-0.0901–0.0816)	-0.03 days	0.92
	White	Reference		
Insurance status	Medicare	-0.0531 (-0.1473–0.0412)	-0.4 days	0.27
	Medicaid	0.0099 (-0.0787–0.0984)	0.08 days	0.83
	Private	-0.1966 (-0.2879 –-0.1053)	-1.4 days	<0.0001
	Other insurance	-0.1073 (-0.3028–0.0881)	-0.8 days	0.28
	Uninsured	Reference		
Teaching status of hospital	Teaching	-0.1420 (-0.2337 –-0.0503)	-1 day	0.003
	Non-teaching	Reference		
Geographic Region	Northeast	0.0643 (-0.0681–0.1968)	0.5 days	0.34
	Midwest	-0.0616 (-0.2884–0.1653)	-0.5 days	0.59
	South	0.0546 (-0.0655–0.1747)	0.4 days	0.37
	West	Reference		

**Table 5:** Summary of results from multivariable logistic regression model examining the impact of septicemia on in-hospital mortality.

Independent Variables		Odds Ratio (95% CI)	p-value
Septicemia	Yes	31.33 (20.25–48.48)	<0.0001
	No	Reference	
Age	Each 1 year increase	1.04 (1.03–1.06)	<0.0001
Sex	Female	0.84 (0.59–1.19)	0.33
	Male	Reference	
Comorbid burden	Each 1 unit increase	1.14 (1.02–1.26)	0.02
Race	Black	1.26 (0.79–2.09)	0.33
	Hispanic	1.08 (0.54–2.15)	0.83
	Asian/Pacific Islander	1.93 (0.92–4.08)	0.08
	Native American	4.58 (1.47–14.22)	0.009
	Other Races	2.59 (1.29–5.18)	0.008
	White	Reference	
Insurance Status	Medicare	0.48 (0.15–1.54)	0.22
	Medicaid	0.37 (0.10–1.38)	0.14
	Private Insurance	0.46 (0.15–1.43)	0.18
	Other Insurance	0.39 (0.10–1.97)	0.25
	Uninsured	Reference	
Teaching Status	Teaching Hospital	1.03 (0.30–3.56)	0.96
	Non-Teaching Hospital	Reference	
Hospital Region	Northeast	1.56 (0.76–3.23)	0.22
	Midwest	1.07 (0.57–2.04)	0.83
	South	1.67 (0.94–2.96)	0.08
	West	Reference	

Results of the multivariable logistic regression model examining the factors associated with development of septicemia are summarized in Table 6. Each one year increase in age was associated with a significantly higher odds for developing septicemia (OR = 1.02, 95% CI = 1.01–1.02, p = 0.0001). Each one unit increase in co-morbid burden was associated with a significantly higher odds for developing septicemia (OR = 1.57, 95% CI = 1.42–1.74, p<0.0001). Sex, type of admission, race, insurance status, teaching status of hospital, and hospital region were not significantly associated with development of septicemia.

**Table 6:** Multivariable logistic regression model for examining factors associated with developing septicemia.

Independent Variables		Odds Ratio (95% CI)	p-value
Age	Each 1 year increase	1.02 (1.01–1.02)	0.0001
Sex	Female	0.98 (0.78–1.22)	0.84
	Male	Reference	
Comorbid burden	Each 1 unit increase	1.57 (1.42–1.74)	<0.0001
Type of admission	Elective	0.77 (0.58–1.03)	0.07
	Emergency/Urgent	Reference	
Race	Black	1.14 (0.89–1.45)	0.30
	Hispanic	0.99 (0.74–1.32)	0.94
	Asian/Pacific Islander	1.04 (0.64–1.69)	0.88
	Native American	0.24 (0.04–1.68)	0.15
	Other Races	1.05 (0.63–1.76)	0.85
	White	Reference	
Insurance Status	Medicare	0.57 (0.17–1.88)	0.35
	Medicaid	1.05 (0.27–4.00)	0.94
	Private Insurance	0.51 (0.15–1.76)	0.28
	Other Insurance	0.88 (0.27–2.90)	0.83
	Uninsured	Reference	
Teaching Status	Teaching Hospital	0.94 (0.40–2.24)	0.89
	Non-Teaching Hospital	Reference	
Hospital Region	Northeast	1.02 (0.67–1.55)	0.93
	Midwest	1.00 (0.68–1.45)	0.98
	South	1.08 (0.69–1.67)	0.74
	West	Reference	

## Discussion

Solid organ transplantation is the preferred therapy for most patients with chronic end-stage organ disease. Currently, renal transplantation is the most frequently performed solid organ transplantation in the United States [7]. Current national estimates of the risk of infection in patients hospitalized primarily for renal transplantation procedure and its impact on outcomes are unclear. Using the largest all payer in-hospital discharge dataset in United States, we show that septicemia of any cause occurred in 2.2% of patients who received renal transplantation and it is associated with higher hospital charges, longer length of stay and increased odds of mortality. After adjustment for multiple patient/hospital level factors, renal transplant recipients who developed septicemia in the post-transplant period had significantly worse outcomes (IHM-odds ratio: 31; 121% increase from mean in LOS; and 83.5% higher HC) compared to their counterparts. Further, of the factors assessed, increasing age (older patients) and co-morbid burden (higher the co-morbidity worse the outcomes) were independent predictors of occurrence of septicemia in this surgical cohort.

In general, advances in surgical techniques, donor matching, immunosuppressive therapy, and perioperative care of the recipient have all contributed to high immediate success rates in renal transplantation [21]. Renal transplant recipient mortality at 90 days, 6 months, 1 year, 5 years and beyond due to variety of causes has been well described in literature and estimated post-transplant survival calculation is now available to guide candidacy selection to optimize outcomes [10, 21,22]. Recipient mortality due to complications, especially infection-related post-operative complications, is viewed as a quality measure in most transplant centers. In the present study, patients who developed septicemia of any cause had a significantly higher risk of mortality compared to their counterparts. The findings from this study further add to the available literature by confirming that bacterial infections are an important cause of complications in the early post-transplant period [23,24].

In the present study, risk factors associated with the occurrence of septicemia included increasing age and higher co-morbid burden. In general, older persons are thought to have a greater susceptibility to infections and associated worse outcomes compared to younger individuals due to variety of factors including immunosenescence [25,26]. Several investigators have described the effect of increasing age on the risk of post operative infections. The relationship is complex, controversial and occasionally contradictory [27,28,29,30,31]. In one large multi center study which included 144,485 consecutive all surgical patients, increasing age (17 to 65 years) was an independent predictor of increased risk of surgical site infection (risk of SSI increased by 1.1%/year, p = 0.002); however, in the same study, at age > 65 years, increasing age independently predicted a decreased risk of SSI (SSI decreased by 1.2% for each additional year, p = 0.008) [27]. In the present study, we show that increasing age is an independent predictor of occurrence of septicemia of any cause. The findings assume importance since septicemia is associated with higher mortality rate in renal transplant patients as shown in this study.

Presence and severity of co-morbid conditions are important predictors of long term outcomes in renal transplant recipients. Of the co-morbid conditions, diabetes mellitus status is one of the four factors used in the calculation of the EPTS [9, 10]. In a single transplant center’s experience of assessing the impact of co-morbid conditions of 715 renal transplant recipients on outcomes, it was revealed that high co-morbidity was associated with an increased risk of mortality in the perioperative period (hazard ratio 3.20, 95% confidence interval 1.32 to 7.78; P = 0.01) as well as at > 3 months after transplantation (HR 2.63; 95% CI 1.62 to 4.28; P<0.001) [32]. Co-morbid measures for use with administrative data such as NIS have been well described [33]. In the present study, we show that higher co-morbidity in renal transplant recipients confers higher risk of occurrence of septicemia in the post-operative period. Co-morbidity as risk factor for occurrence of sepsis has been well described in large epidemiological studies [34,35].

Previous studies have shown that renal transplantation not only confers better quality of life but also cost effectiveness in comparison to maintenance dialysis [6,36,37,38]. This benefit is apparent in long term survivors of the transplantation procedures [39,40]. To our knowledge, the economic impact of infectious complications in patients hospitalized for renal transplantation has not been adequately reported. We address this knowledge gap to an extent. In the present study, we show that the occurrence of septicemia of any cause in renal transplant recipients is associated with increased hospital charges and longer duration of stay in hospital.

To our knowledge, the present study is the largest cohort of renal transplant recipients whose risk of infection during the hospitalization for transplantation is assessed. Comparative studies are limited to single centers, smaller numbers or older data. The NIS represents experiences beyond single centers and hence the results from this study are generalizable. Identification of risk factors for developing septicemia (increasing age and co morbidity) in this unique population may enable optimization of outcomes. The findings in our study assume importance given the availability of alternative, albeit sub-optimal treatment modalities such as dialysis and the limited supply of organs. The present study also highlights the significantly higher hospital charges and length of stay associated with septicemia in this transplant cohort.

Our study has several limitations. Its retrospective nature precludes establishing true cause and effect relationships; nevertheless, the associations shown in this study (increased hospital charges, longer length of stay, and higher risk of mortality in patients with septicemia) appear to have merit since it is widely known that infection and septicemia lead to higher risk of mortality in hospitalized surgical population [41]. Another limitation to our study arises from the use of large secondary hospital discharge datasets with its potential for billing and coding inaccuracies that may exist among and within the hospitals that provide data to this nationalized sample. However, robust quality measures in collecting and reporting data ensure minimizing any systematic variations in coding practices [16]. The utility of the NIS datasets has been widely recognized as evidenced by numerous publications arising from these datasets [16]. The NIS dataset does not contain patient level physiologic (example heart rates, blood pressures) or biological data (including lab values), precluding us from assessing the impact of severity of septicemia or other organ dysfunction on outcomes. To offset this limitation, we used extensive ICD 9 CM codes to identify the different types of septicemia (with or without organ dysfunction), as well as septicemia caused by various organisms. The use of ICD 9 CM codes to identify sepsis has been well validated with a positive predictive value of 88.9%, sensitivity of 87.7%, specificity of 98.8% and negative predictive value of 98.6% [42,43,44]. Further, the utility of using ICD 9 codes in the diagnosis of septicemia in renal transplant recipients has also been reported in prior publications [24]. In addition, the nature of the dataset precludes us from estimating the prevalence and use of antibiotics (anti-infective’s), appropriateness, timing, and/or resistance pattern to antibiotics which have all shown to influence outcomes. In the scenario wherein optimal use of anti-infective’s was assured then the findings in our study could likely be an underestimate and would highlight the need for further optimizing the infection preventive programs. We used hospital charges as one of the outcome variables. Hospital charges typically refers to the $ amount levied at the time of discharge. This is not a true estimate of the actual costs involved in treatment. Omitted variable bias has to be accounted for before interpreting the findings in our study. In the present study we examined the charges levied by the hospital only (for the index hospitalization). A considerable population in this study who developed septicemia were discharged to long term care facilities (example: skilled nursing facilities) and home health care. In addition, the present study does not capture the impact of disability, loss of productivity, long term outcomes and the impact on mental health of both the living donor and recipient all which clearly underestimates the findings in our study.

Health care associated infections, including those related to surgical procedures, are a major cause of morbidity and mortality throughout the world. The impact of adverse outcomes in fresh renal transplant recipients has enormous consequences for the patients, families (especially the living donor), providers and the health care system. Although, the overall prevalence of septicemia appears to be low in this study, the outcomes associated with it are not in-significant. The present study highlights the need to further strengthen the existing infection preventive programs, universal implementation of surgical safety check lists [41], ensuring mandatory compliance with well established recommendations from health governing agencies [45] and optimal patient risk assessment and modification if possible.

## Conclusions

Despite advances in medical/surgical care, septicemia occurred in 2.2% of patients having renal transplantation procedures in United States and is associated with poor outcomes including higher hospital charges, longer length of stay in hospitals, and increased risk for in-hospital mortality. Older patients and those with higher co-morbid burden are at an increased risk of developing septicemia.
